# Secrets of virtuoso: neuromuscular attributes of motor virtuosity in expert musicians

**DOI:** 10.1038/srep15750

**Published:** 2015-10-27

**Authors:** Shinichi Furuya, Takanori Oku, Fumio Miyazaki, Hiroshi Kinoshita

**Affiliations:** 1 Department of Information and Communication Sciences, Sophia University, Japan; 2 Institute for Music Physiology and Musicians’ Medicine, Hanover University of Music, Drama and Media, Germany; 3 Department of Engineering Science, Osaka University, Japan; 4 Graduate School of Medicine, Osaka University, Japan

## Abstract

Musical performance requires extremely fast and dexterous limb movements. The underlying biological mechanisms have been an object of interest among scientists and non-scientists for centuries. Numerous studies of musicians and non-musicians have demonstrated that neuroplastic adaptations through early and deliberate musical training endowed superior motor skill. However, little has been unveiled about what makes inter-individual differences in motor skills among musicians. Here we determined the attributes of inter-individual differences in the maximum rate of repetitive piano keystrokes in twenty-four pianists. Among various representative factors of neuromuscular functions, anatomical characteristics, and training history, a stepwise multiple regression analysis and generalized linear model identified two predominant predictors of the maximum rate of repetitive piano keystrokes; finger tapping rate and muscular strength of the elbow extensor. These results suggest a non-uniform role of individual limb muscles in the production of extremely fast repetitive multi-joint movements. Neither age of musical training initiation nor the amount of extensive musical training before age twenty was a predictor. Power grip strength was negatively related to the maximum rate of piano keystrokes only during the smallest tone production. These findings highlight the importance of innate biological nature and explicit training for motor virtuosity.

Motor virtuosity represents extremely fast and dexterous motor actions of highly skilled individuals, such as musicians, athletes, and surgeons, which has fascinated people worldwide for centuries^1^. To unlock its secrets, a large body of literature investigated the attributes of the production of nimble, precise, and complex movements. For example, the temporal precision of finger movements in musicians correlated with the age of musical training commencement and the amount of intensive musical training^2^,^3^,^4^. In contrast, recent studies demonstrated a genetic predisposition for fine motor control in musicians^5^,^6^. However, there are two major limitations of previous studies. First, these studies commonly evaluated the performance of a simple motor task, such as single finger tapping, which largely differs from the skilled movements that typically require the coordination of motion across multiple joints. Second, most of the studies investigated the temporal accuracy but not the speed of the rhythmic movements. For example, the tapping speed in musicians was not correlated to the age of commencing musical training or the amount of total musical practice^3^,^7^. Accordingly, little is understood about the biological mechanisms that underlie the production of fast movements of naturalistic motor tasks, such as sports and musical performance.

The maximum movement speed at the limb endpoint (i.e., fingertip, instrument) does not necessarily correlate with movement speed at all joints in multi-joint upper-limb movements during musical performance, such as repetitive strikes of piano keys^8^,^9^,^10^ and drum pad^11^,^12^. For example, an individual difference in the maximum rate of the alternate strikes of two keys between pianists correlated with the maximum speed of elbow rotation but not finger rotation^9^. This result suggests a non-uniform relationship of the maximum speed between the fingertip and individual joints. Furthermore, musical performance constrains muscular force production in loudness of tone production, in contrast to most fast movements, such as ball-throwing^13^,^14^ and sprint running^15^, where muscular strength determines the maximum movement speed. Therefore, musical performance may provide a unique opportunity to better understand the specialized motor skills that are required to produce fast naturalistic multi-joint movements, unlike simplistic motor tasks and sports activities.

The present study aims at identifying attributes of inter-individual differences in the maximum rate of repetitive piano keystrokes in skilled pianists. The repetitive strikes of two keys with the thumb and little finger were used in this study because our previous studies confirmed that a skillful coordination of multiple joints from the shoulder to the finger is required for movement production^10^,^16^,^17^. These movements are characterized by variations of patterns of multi-joint coordination in relation to tempo and loudness^8^. The present study further identified predictors of the maximum keystroke rate at a variety of loudness because the joint coordination pattern differs depending on loudness^10^ and more rapid movements result in louder sounds, which demands more to produce rapid movements without altering loudness. We assessed musical training history, such as the age of musical training initiation and the amount of intensive musical practice before age twenty, anatomical characteristics, and neuromuscular functions at individual body portions, such as motor agility and muscular strength. These factors were chosen because most of them were widely investigated in previous studies of simplistic motor tasks^18^. An understanding of the predictors sheds light on the biological mechanisms subserving virtuosic motor performance and provides a perspective on which principled training strategies may be built.

## Results

Twenty-four pianists, who ranged from winners of international piano competitions to amateur players, were asked to strike two piano keys repetitively with their thumb and little finger as fast as possible at four different loudness levels. The maximum rate of the piano keystrokes was computed based on key-position data measured using a high-speed camera. We further evaluated neuromuscular functions, such as the tapping rate of the finger, wrist, and elbow, the maximum force of the individual muscles of the upper-limb, power-grip force and the history of musical training, such as the age of starting piano playing and total hours of piano practice until age twenty, as possible predictors. These factors were used as independent variables in a stepwise multiple regression analysis to identify potential predictors of inter-individual differences in the maximum rate of piano keystrokes across the players at each loudness level. We used a stepwise regression to reduce the number of predictor variables because of a relatively small sample size compared to the number of independent variables (a variable selection criterion; p < 0.05). The prediction model of the maximum keystroke rate based on these selected variables was then rebuilt using a generalized linear model (GLM).

Table 1 summarizes the maximum, minimum, mean, and standard deviation values of the maximum rates of the fastest and repetitive multi-joint upper-limb piano keystrokes at four loudness levels, neuromuscular variables, anatomical characteristics, and history of musical training across all pianists. The maximum rate of piano keystrokes varied from 6.0 to 7.8 Hz among the pianists. A one-way repeated-measures ANOVA determined a significant main effect of loudness on the maximum rate (F(3,69) = 5.01, p = 0.003, η^2^ = 0.03). The loudness effect indicates that pianists played faster at louder tone production. The mean and standard deviation of the maximum rate of piano keystrokes of each participant was computed for each loudness levels, and a correlation coefficient between these two variables was computed across all participants to further assess whether the faster strikes of pianists were merely due to lower movement accuracy according to a speed-accuracy tradeoff^19^. The results confirmed no correlation between the maximum rate (i.e., mean of the maximum rate of the keystrokes) and rhythmic inaccuracy (i.e., standard deviation of the maximum rate of the keystrokes) of repetitive piano keystrokes (r = 0.12, 0.30, 0.17, and −0.18, and p = 0.58, 0.16, 0.43, and 0.41, for p, mp, mf, and f, respectively), which excluded a confounding effect of the speed-accuracy tradeoff for inter-individual differences in keystroke rate.

**Figure 1:**
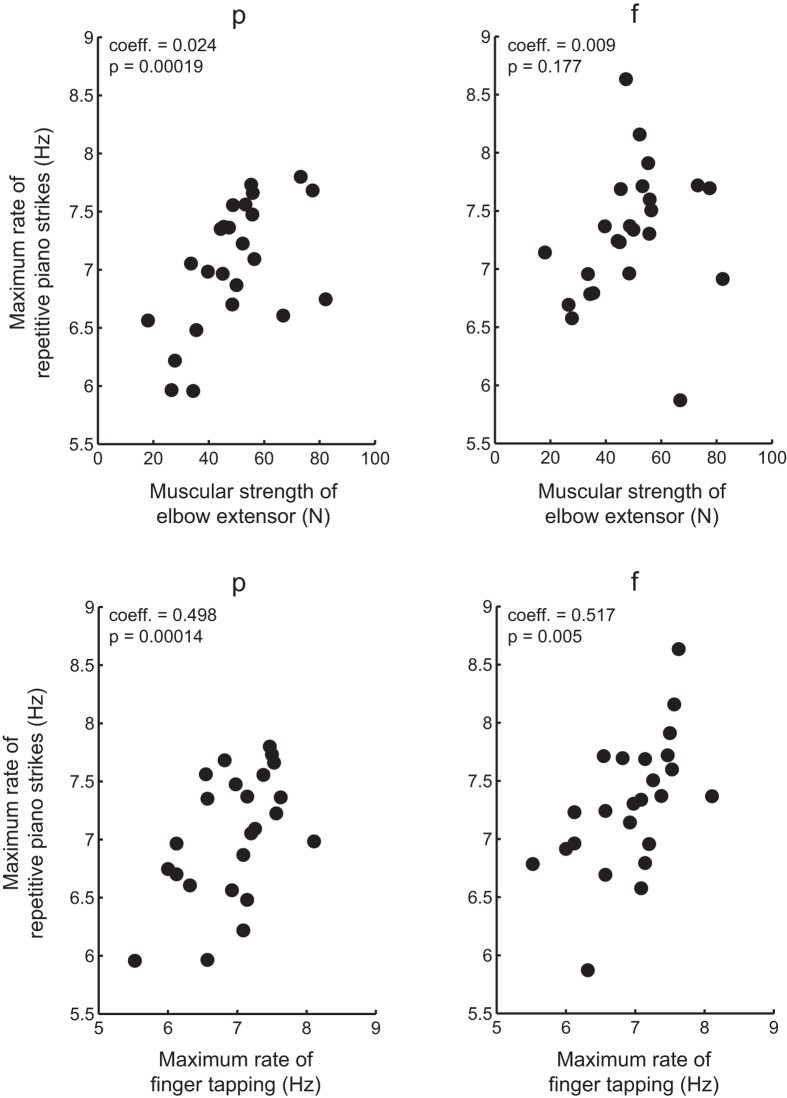
A scatter plot displaying the relationship between the maximum rate of repetitive piano keystrokes (y-axis) and each of the maximum forces exerted by the elbow extensor muscles (top panel) and the maximum rate of finger tapping (bottom panel) at p (left panel) and f (right panel) loudness. Each dot represents a single piano player. “coeff.” and “p” indicate partial regression coefficients and p values derived from stepwise multiple regression analyses, respectively.

A stepwise multiple regression analysis was performed for each loudness level to identify a small set of predictors of the maximum rate of repetitive piano keystrokes. The analysis identified that the maximum muscular forces for extension and flexion were not significant predictors at any of the loudness levels (p > 0.05), except for the elbow extension muscular force at p, mp, and mf (Table 2). Concerning the tapping rate, only the finger was identified as a significant predictor at all loudness levels (Table 2), but neither the wrist nor elbow was significant (p > 0.05). Figure 1 illustrates a positive relationship between the maximum keystroke rate and each of the elbow extensor forces and single-finger tapping rate. Notably, the only significant variable at the loudness of f was confined to the maximum rate of single finger tapping. Grip force was also added as a significant attribute at the loudness of p (i.e., the smallest tone). Hand span, starting age of piano playing, or accumulated hours of piano practice before age twenty were not significant in the stepwise regression analysis at all loudness levels (p > 0.05). Notably, the selected variables were robust against the direction of the stepwise analysis (i.e., either forward or backward). The R^2^ and p values of the regression model were 0.68 and 3.49 × 10^−5^ at p, 0.57 and 1.44 × 10^−4^ at mp, 0.45 and 0.002 at mf, and 0.31 and 0.005 at f, respectively.

A GLM was developed at each loudness level using the variables selected by the stepwise regression. We compared models under the assumption of different data distributions (e.g., Gaussian, Gamma, and inverse-Gaussian) because of the lack of prior information on data distribution and relatively small sample size. The comparison was performed based on AIC computed by GLM (i.e., smaller AIC indicates a better model). The results demonstrated that the best regression model of the maximum rate of piano keystrokes was obtained when assuming the data distribution as Gaussian at p (AIC = 20.82), Gamma at mp (AIC = 24.65), inverse-Gaussian at mf (AIC = 34.04), and Gaussian at f (AIC = 37.50). Table 2 summarizes the coefficients and corresponding p values of the individual independent variables. We further obtained the confidence interval (CI) of the individual GLM coefficients using bootstrap sampling over 1000 times for each of the loudness levels (Table 2). The results confirmed the robustness of the sign of the individual coefficients between the 2.5% and 97.5% CI.

Previous studies assessed relationships between finger tapping rate and the amount of practice and the starting age of musical training. Therefore, we further assessed correlations between the maximum rate of finger tapping and these two variables. The results demonstrated that neither the total amount of practice duration (r = 0.03, 0.02, 0.04, and p = 0.90, 0.92, 0.84 for the finger, wrist, elbow) nor the starting age of musical training (r = 0.24, 0.24, 0.17, and p = 0.26, 0.26, 0.42 for the finger, wrist, elbow) significantly correlated with the tapping rate.

## Discussion

Previous studies have focused on investigating the precise temporal control of a simple motor task. In contrast, the present study assessed predictors of individual differences in the maximum rate of repetitive piano keystrokes, which involves naturalistic multi-joint upper-limb movements, among twenty-four pianists. Our results demonstrated that two factors predicted individual differences in the keystroke rate: the maximum rate of single finger tapping and the maximum strength of elbow extensor muscles. The fastest tapping rate was used to assess movement quickness at a target body part, such as finger, wrist, and elbow^20^,^21^,^22^. The correlation between the finger tapping rate and maximum rate of repetitive piano keystrokes implies a shared motor control mechanism between these tasks. The lack of correlation with the wrist and elbow tapping rate further suggests no predominant role of the ability to move these proximal joints quickly in the production of fairly fast multi-joint piano keystrokes.

Previous studies found changes in the maximum rate of repetitive finger tapping with training in musically untrained individuals^23^,^24^. For example, one year of intensive musical training in children without formal musical education facilitated their finger tapping rate, and this facilitation was associated with an increase in the grey matter volume of the motor cortex^25^. In contrast, the tapping rate in musicians was not correlated with the starting age of musical training or the total duration of intensive musical training^3^,^7^. Our results replicated these findings; no correlation between the finger tapping rate and the amount of practice was observed in expert pianists, which suggests a ceiling effect of extensive training. Possible factors that influence finger movement agility in trained musicians include genetic predisposition^5^,^6^ and the quality of practice.

The second factor was the maximum strength of the elbow extensor muscles. In our previous kinematic study, fast repetitive keystrokes of two keys simultaneously were characterized by tempo-dependent involvement of the elbow extensor muscles^8^. At slow tempo, elbow extension for striking keys was driven by gravity but not by elbow extensor muscles^17^. Force production was substituted by elbow extensor muscles as the tempo increased^8^ because a large acceleration cannot be produced by the gravitational force in fast cyclical arm movements. Faster cyclical arm movements also require larger muscular compensation for the inter-segmental dynamics that originate from movements at adjacent joints^26^. Therefore, the voluntary force production of elbow extensor muscles plays a role in the production of the large acceleration and deceleration that are necessary for the quick reversal of arm movement direction and compensation for the large inter-segmental dynamics. The compensation for large inter-segmental dynamics may explain the lack of a significant association of elbow tapping speed with the maximum rate of the piano keystrokes. Strength training of the elbow extensor muscles may be an effective method for performers and teachers to achieve faster playing.

Notably, a predictor of the maximum rate of piano keystrokes was limited to the finger-tapping rate when eliciting the loudest tones. Our previous kinematic study found a larger increase in finger flexion velocity with tempo at louder tones during repetitive piano key strikes^8^, which is consistent with the present study. Repetitive piano keystrokes forcefully involve the continuous production of large acceleration and deceleration of limb movements. Movement of the proximal limb segment cyclically is energetically costly because the large moment of inertia requires the production of a large kinetic energy. Large acceleration of the proximal segment produces large inter-segmental dynamics at the distal segment. Therefore, compensation for this perturbing dynamic by the distal muscles is physiologically costly.

The power grip force negatively correlated with the maximum rate of repetitive piano keystrokes only when eliciting the smallest tones, which indicates faster keystrokes in pianists with smaller grip force. Even a small deviation of the key-striking force yields a large change in loudness when eliciting a small piano tone^27^. Precise movement control increases the amount of coactivation of antagonistic pairs of muscles, which elevates muscular stiffness and lowers movement variability^28^,^29^. Stronger muscles may produce a greater stiffness at the finger muscles for precise motor control, which may impede the production of fast movements.

Notably, several factors were not identified as significant predictors of the rate of repetitive piano keystrokes. For example, the amount of accumulated practice hours before age twenty (i.e., deliberate practice) was reported as a predictor of the performance of highly skilled movements^7^. In contrast, the current study indicated that the repetition of mere practice does not provide extremely fast piano keystrokes. Genetic factors should be examined in future studies. For example, muscle fiber composition (i.e., slow and fast fibers), which is genetically defined to some extent^30^ , may predict the rate of keystrokes. Previous studies investigated deliberate practice using a simplistic motor action (e.g., finger tapping), but the current motor task involved the skillful coordination of motions across multiple joints. Another possible predictor of individual differences in maximum keystroke rate is some specialized kinematic and kinetic pattern of spatio-temporal coordination of movements^10^, which may depend on the amount and quality of piano practice and teacher instructions. Therefore, motor function and motor skill are better argued distinctly with respect to deliberate practice.

## Methods

### Participants

Twenty-four pianists (17 females, age 19 to 50 years old, all right-handed) participated in the experiment. Eighteen pianists majored or had majored in piano performance at music conservatories, and eleven of these pianists won prizes at international piano competitions, such as the Queen Elisabeth International Music Competition and Concours International Marguerite-Long-Jacques-Thibaud. Six pianists had not formally studied in music conservatories, but privately studied with piano teachers and practiced piano daily. Participants were recruited to minimize the inclusion of pianists with the same educational background (e.g., same teacher, same music conservatory). The local ethics committee of Osaka University approved the experimental protocol, and all participants gave informed consent prior to the experiment. The experiment was performed according to the Declaration of Helsinki.

### Experimental procedure

The present study began with experiments that evaluated history of musical training, anatomical characteristics, and neuromuscular functions. Each participant was asked their age, starting age of piano playing, and accumulated practice hours before age twenty^7^. Hand span was also measured as the distance between the tips of the thumb and little finger with the hand maximally opened. The participant performed a maximum voluntary contraction (MVC) for the flexion and extension of the finger, wrist, elbow, and shoulder of the right upper limb (i.e., six trials). Each subject was verbally encouraged to exert maximal force for three seconds at a designated joint angle against a force transducer that was mounted on a table while sitting in a chair with an upright posture. The finger and wrist joints were kept at 180 degrees (i.e., the neutral position), the elbow was kept at 90 degrees, and the shoulder was kept at 0 degrees (i.e., the neutral position). These joint angles were selected to simulate the geometrical configuration of the upper limb during piano playing. The joints, except for the joint responsible for force production, were immobilized using a splint or Velcro strap, so that the measured force precisely reflected the force exerted solely by the muscles surrounding the target single joint. The force transducer contacted the proximal phalanxes of the middle finger during each finger flexion and extension, the metacarpal bones (40 mm from the wrist joint center) during each wrist flexion and extension, the forearm (150 mm from the elbow joint center) during each elbow flexion and extension, and the upper-arm (150 mm from the shoulder joint center) during each shoulder flexion and extension. The transducer had a measurement range of 0–300 N within a 1% error in linearity, a resolution of 0.2 N, and a natural frequency of DC-1 kHz when unloaded (TEC Gihan Co.). The force signal was amplified using a strain-gauge amplifier (Kyowa Co.) and sampled using a 12-bit analog-to-digital converter at a frequency of 900 Hz. Power grip strength of the right hand was measured as the maximum grip using a grip dynamometer. The maximum movement rate of single-joint tapping was measured for each finger (middle finger), wrist, and elbow of the right upper limb by asking each participant to tap the force transducer as fast as possible. The tapping performance of a certain joint involved the immobilization of the remaining two joints using a splint or Velcro. The upper limb and trunk posture was the same as the postures during the maximum force production.

Following the aforementioned three experiments, each participant was asked to strike two piano keys (E3 and C4) of an upright acoustic piano (YAMAHA U-1) with the thumb and little fingers simultaneously and repetitively as fast and accurately as possible at four loudness levels. The loudness level was determined according to the peak key-descending velocity, which is linearly scaled with loudness (p: 0.18 m/s, mp: 0.21 m/s, mf: 0.24 m/s, f: 0.27 m/s)^27^. The two keys were separated from each other by 112.5 mm. We chose the repetitive chord keystrokes because this task is frequently used in virtuosic musical pieces, and we previously confirmed the involvement of all joints of the upper limb during this task^8^. Thirty successive keystrokes were performed for each loudness level. Key descending movements were measured using a high-speed camera (Hamamatsu Photonics co.) that was located adjacent to the piano at the sampling frequency of 900 Hz.

### Data analysis

A stepwise multiple regression analysis was performed at each loudness level to determine the attributes of individual differences in the maximum movement rate of repetitive multi-joint upper limb piano key strikes across pianists. We used stepwise analysis as a tool for choosing a small set of independent variables as possible predictors and used these selected variables to develop the optimal GLM because of the large number of independent variables relative to the sample size. The stepwise regression analysis was run in both forward and backward directions to ensure that the selected variables were the same regardless of the direction of the analysis. The dependent variable was the rate of the fastest repetitive piano keystrokes, which was computed as the reciprocal of the mean inter-keystroke interval across thirty strikes for each pianist. The inter-keystroke interval was computed based on the time-varying data of the key vertical position. Independent variables related to neuromuscular functions included the maximum rate of single-joint tapping using the finger, wrist, and elbow (i.e., tapping rate) and the force exerted during MVC for the flexion and extension of the finger, wrist, elbow, and shoulder joints (i.e., maximum muscular force). The maximum tapping rate was defined as the reciprocal of the mean inter-tap interval derived from the time-varying fingertip force data, and the maximum muscular force was computed as the mean value during the MVC task. Variables related to the history of musical training and anatomical characteristics were also included in the same regression analysis. The fundamental data analyses and stepwise regression analyses were performed using MATLAB (Mathworks co.).

The GLM was developed at each loudness level using the independent variables that were selected from the stepwise multiple regression analysis. GLMs that assumed different distribution functions, including Gaussian, Gamma, and inverse-Gaussian, were developed because of the lack of prior information on data distribution and compared according to Akaike’s information criterion (AIC). Therefore, the optimal model exhibited the smallest AIC value. Bootstrap resampling over 1000 times was run for the individual GLM to further obtain the confidence interval of coefficients of the model. GLM and bootstrapping was performed using R (ver. 3.1.1).

## Additional Information

**How to cite this article**: Furuya, S. *et al.* Secrets of virtuoso: neuromuscular attributes of motor virtuosity in expert musicians. *Sci. Rep.* **5**, 15750; doi: 10.1038/srep15750 (2015).

## Figures and Tables

**Table 1:** Mean, SD, maximum, and minimum values of the variables evaluated across participants.

	Max	Min	Mean	SD
Max. rate of piano keystrokes (Hz) p	7.8	6.0	7.0	0.5
Max. rate of piano keystrokes (Hz) mp	8.1	6.1	7.1	0.5
Max. rate of piano keystrokes (Hz) mf	8.6	6.1	7.2	0.6
Max. rate of piano keystrokes (Hz) f	8.6	5.9	7.3	0.6
Max. muscular force: finger-flx (N)	100.5	28.2	63.9	15.1
Max. muscular force: finger-ext (N)	66.7	12.3	26.5	11.6
Max. muscular force: wrist-flx (N)	135.2	46.9	72.9	23.6
Max. muscular force: wrist-ext (N)	92.9	30.5	63.5	16.6
Max. muscular force: elbow-flx (N)	208.6	43.8	111.6	37.5
Max. muscular force: elbow-ext (N)	82.2	18.1	48.9	15.8
Max. muscular force: shoulder-flx (N)	103.7	19.9	41.1	18.1
Max. muscular force: shoulder-ext (N)	86.8	23.4	44.9	16.0
Tapping rate: finger (Hz)	8.1	5.5	6.9	0.6
Tapping rate: wrist (Hz)	7.8	5.1	6.9	0.7
Tapping rate: elbow (Hz)	8.3	4.6	6.6	0.9
Hand span (mm)	226	178	0.2	0.0
Maximum grip force (N)	480.2	205.8	306.4	75.4
Age of starting piano (yrs)	13	3	4.9	2.4
Accum. practice hours (hrs)	27740	1825	14204.6	7343.4

“Max. rate of piano keystrokes “ indicates the maximum rate of the repetitive piano keystrokes. “Tapping rate” indicates the rate of the fastest single-joint tapping. “Max. muscular force” indicates force exerted during the maximum voluntary contraction (MVC). “Accum. practice hours” indicates the sum of total piano practice before age 20. p, mp, mf, and f indicates piano, mezzo-piano, mezzo-forte, and forte, respectively.

**Table 2:** Results of a generalized linear model (GLM) and bootstrapping for the maximum rate of the piano keystrokes.

	Finger tapping rate	Maximum elbow extension force	Grip force
loudness			
p			
coefficient	0.502	0.027	−0.025
CI (2.5%)	0.244	0.015	−0.047
CI (97.5%)	0.714	0.038	−0.007
p-value	2.56E-04	5.38E-05	0.031
mp			
coefficient	−0.012	−2.55E-04	
CI (2.5%)	−0.019	−4.66E-04	
CI (97.5%)	−0.006	−7.15E-05	
p-value	1.05E-04	0.014	
mf			
coefficient	−0.003	−7.59E-05	
CI (2.5%)	−0.006	−1.40E-04	
CI (97.5%)	−0.002	−1.33E-05	
p-value	0.001	0.029	
f			
coefficient	0.517		
CI (2.5%)	0.266		
CI (97.5%)	0.998		
p-value	0.005		

“E-n” indicates “× 10^–n^”. (e.g. 2.56E-04 indicates 2.56 × 10^−4^). “coefficient” indicates the partial regression coefficient computed by GLM. CI: confidence interval of the individual coefficients derived from bootstrapping. p, mp, mf, and f indicates piano, mezzo-piano, mezzo-forte, and forte, respectively. Note that a stepwise regression included “grip force” only at p, and “maximum elbow extension force” at p, mp, and mf.
